# Book Review: Payback: Why We Retaliate, Redirect Aggression, and Take Revenge

**DOI:** 10.3389/fpsyg.2016.02062

**Published:** 2017-01-09

**Authors:** Nereida Bueno-Guerra

**Affiliations:** Department of Psychology and Clinical Psychobiology, University of BarcelonaBarcelona, Spain

**Keywords:** payback, retaliation, redirected aggression, revenge

Regrettably, revenge is in the spotlight. Just to mention three recent examples, the news^1^ (15th November 2015) defined the bombs dropped by France on Islamic State Jihadi training camp after the Paris shootings as “revenge.” Only 2 days after, president Putin literally claimed on TV that “revenge is unavoidable”^2^ (17th November 2015) toward the Syrian rebels responsible for the Russian jet downed the day before. More recently, the Iranian supreme Ayatollah leader vowed for “divine revenge”^3^ (3rd January 2016) on Saudi Arabia after the execution of one of their prominent clerics. Such an intricate international scenario of aggression demands reflection on the factors involved in human vengeance as well as on its causes and solutions. That is why I recover *Payback* from the shelves, a 209 pages book written by a married couple (he an evolutionary biologist, she a psychiatrist) that matches all the current breaking news on revenge despite dating back from 2011.

Firstly, the subtitle of the book is not pointless. There is some difficulty to find a subtle English native differentiation between the what-so-called “three Rs” (retaliation, revenge, and redirected aggression). In this sense, the authors provide clarifying definitions in the very first chapter (pp. 4–5):

“*When one being hurts another, several things may happen. Sometimes, the pain is immediately reflected back onto the perpetrator. This is retaliation. It is prompt and straightforward. (…) Then there is revenge. (…) The response is delayed–often for a long while and with much prior contemplation. (…) The strangest form of payback (…) is redirected aggression (…)–the targeting of an innocent bystander in response to one's own pain and injury.”*

However, this exercise of clarification with the core terms of the book unfortunately vanishes across the book due to lack of scientific support to the ideas argued. This is because *Payback* grounds on a personal experience more than on a defined line of research. In the Preface, the authors confess that the boost to write a book like this appeared after a marital problem coming from how they had transmitted their political commitment to their children. The reading of a Buddhist essay eventually helped them (and it was a crush indeed provided that one of the authors published a book exclusively focused on Buddhism afterwards, Barash, 2013). Their personal revelation consisted of writing a book based on how to avoid “pain passing” (sic.) and fostering forgiveness. That is why by reading the Preface the audience learns that *Payback* is not going to be a regular monograph on aggression offering different theoretical backgrounds plus empirical studies but a compilation of anecdotes on aggression plus a Buddhist proposal on forgiving. Then, which are the advantages and disadvantages of reading *Payback* for a scientific audience?

Regarding advantages, one of the book's strongest points is the authors' ability to shred payback from different disciplines whereas doing an exquisite labor of documentation. The eclectic index provides with first contact to different fields that may help any researcher interested in aggression to rethink their line of research from a renewed perspective. Chapter 1 lists popular examples (i.e., a Tim Burton's film, conflicts in Bosnia, Gaza) to illustrate the differences among the three Rs. Chapter 2 discards retaliation and revenge from the evolutionary perspective and focuses on redirected aggression in non-human animals. Chapter 3 refers to victimization (i.e., bullying, sexual abuse) and scapegoating with references to religion, literature, and economics. Chapter 4 mixes Sociology and History examining different group conflicts (i.e., tribes, World War I). Chapter 5 reviews mythology, Ancient Greek theater (i.e., Oresteia, Medea), and classical authors (i.e., Shakespeare, Dostoevsky). Chapter 6 separates gut need for revenge with social need for impartial justice stressing the victims' feeling of satisfaction. Chapter 7 runs through the visions of different religions and disciplines to overcome pain passing. Finally, the one-page conclusion (Chapter 8) proposes somewhat as a self-pray to focus on decreasing pain.

The main contribution of this book for the current course of international events mentioned at the beginning of this review is found in Chapter 7. It offers a fully detailed description of how different perspectives (Islam, Christianity, and Judaism among others) deal with aggression and conciliation. Of course, this chapter can only work as an introductory approach for those researchers interested in intervention against aggressive behavior from the point of view of cultural background. However, it fulfills the expectations of those interested in learning the motivations and needs that the counterparts of conflicts might harbor.

Regarding disadvantages, covering payback too widely makes extremely difficult to find a concrete target of readers, as already mentioned by Sung (2011). Is *Payback* for evolutionary biologists or comparative psychologists? It lacks of the background that could be better found in McCullough (2008). Is *Payback* for literature or religious scholars? Wild Justice by Susan Jacoby (1983) might be a better first option instead. This book may be eligible for students in social sciences as well as for a general audience interested in reciprocity and forgiveness that enjoy continuous references to folk culture, cases, and anecdotes.

In addition, a book inspired by a personal experience makes the authors' subjective view perfectly recognizable across the book. This is especially evident in their last chapter, close to a self-help book (p. 199):

“*When evaluating alternative actions, I will ask myself whether each is likely to increase or decrease the total amount of pain in the world, and I will always choose the latter”*

In conclusion, Payback succeeds at pinpointing aggression and conciliation in a whole array of fields theoretically-related and from different cultural perspectives. This broad picture may be useful for researchers so that it promotes the collaboration between different disciplines as well as it helps to elucidate the non-univocal causes for the current political scenario.

## Author contributions

The author confirms being the sole contributor of this work and approved it for publication.

## Funding

The author grants a FPU scholarship from the Spanish Ministry of Education. Ref. FPU12/00409.

### Conflict of interest statement

The author declares that the research was conducted in the absence of any commercial or financial relationships that could be construed as a potential conflict of interest.

## Footnotes

^1^France drops 20 bombs on Islamic State jihadi training camp in revenge for Paris shootings, MIRROR. Available online at: http://www.mirror.co.uk/news/world-news/france-drops-20-bombs-islamic-6838318 (accessed February 14, 2016).

^2^Vladimir Putin: “Revenge is unavoidable,” CNN. Available online at: http://edition.cnn.com/videos/world/2015/11/17/putin-syria-airstrike-metrojet-matthew-chance-lkl-newsroom.cnn (accessed February 14, 2016).

^3^Iran: Saudis face “divine revenge” for executing al-Nimr, BBC News. Available online at: http://www.bbc.com/news/world-middle-east-35216694 (accessed February 14, 2016).
